# Auxins and Cytokinins Regulate Phytohormone Homeostasis and Thiol-Mediated Detoxification in the Green Alga *Acutodesmus obliquus* Exposed to Lead Stress

**DOI:** 10.1038/s41598-020-67085-4

**Published:** 2020-06-23

**Authors:** Alicja Piotrowska-Niczyporuk, Andrzej Bajguz, Urszula Kotowska, Elżbieta Zambrzycka-Szelewa, Aneta Sienkiewicz

**Affiliations:** 1 University of Bialystok, Faculty of Biology, Department of Plant Biology and Ecology, Ciolkowskiego 1J, 15-245 Bialystok, Poland; 2 University of Bialystok, Faculty of Chemistry, Department of Analytical and Inorganic Chemistry, Ciolkowskiego 1K, 15-245 Bialystok, Poland; 3 Bialystok University of Technology, Department of Agri-Food Engineering and Environmental Management, Wiejska 45A, 15-351 Bialystok, Poland

**Keywords:** Plant hormones, Plant physiology, Plant stress responses, Plant sciences

## Abstract

Phytohormones, such as auxins and cytokinins, take part in the integration of growth control and stress response, but their role in algal adaptation to heavy metal remains to be elucidated. The current research indicated that lead (Pb), one of the most toxic metals in nature, causes severe depletion of endogenous cytokinins, auxins, and gibberellin and an increase in abscisic acid content in the green alga *Acutodesmus obliquus*. Exogenous auxins and cytokinins alleviate Pb toxicity through the regulation of the endogenous phytohormones’ levels. Exogenously applied auxins provoked the coordinated activation metal tolerance mechanisms leading to the increase in phytochelatin synthase activity and accumulation of phytochelatins and their precursors, which are essential for Pb sequestration. On the other hand, phytochelatin synthesis decreased in algal cells treated with cytokinins. Significant changes in the levels of low molecular weight metabolites, mainly involved in metal chelation and glutathione synthesis pathway under the influence of phytohormones in algal cells growing in the presence of Pb stress, were observed. This is the first report showing that auxins and cytokinins are important regulatory factors in algal adaptation strategies to heavy metal stress based on thiol-mediated compounds and the maintenance of phytohormone homeostasis.

## Introduction

Contamination of aquatic ecosystems with lead (Pb) is a matter of great globall concern. Freshwater in many areas may contain up to 0.1 µM Pb. However, anthropogenic activity, including manufacturing, mining, smelting, and the continued use of paint and aviation fuel with Pb leads to increases in concentrations of this heavy metal in water bodies, up to 2000 uM^1,2^.

Pb is highly toxic for algae living in freshwater ecosystems, as it is not an essential nutrient. Although green microalgae such as *Acutodesmus obliquus* are less morphologically advanced than vascular plants, they have many similar biochemical processes and responses to abiotic stress, which could potentially be under phytohormone control^3,4^. The presence and/or functions of auxins (i.e., indole-3-acetic acid, IAA; indole-3-butyric acid, IBA; phenylacetic acid, PAA) and cytokinins (i.e., *trans*-zeatin, *t*Z; kinetin, Kin; *N,N’*-diphenylurea, DPU) were confirmed in different microalgal species from *Chlorella*, *Scenedesmus* and *Acutodesmus* genus^5–9^. These phytohormones are the most essential endogenous molecules for modifying physiological and molecular reactions and are critically required by the plant for its survival under heavy metal stress. They act as chemical messengers and, under a complex regulation, allow plants to sustain abiotic stresses such as excess heavy metal ions. Although some papers have demonstrated that auxins and cytokinins can alleviate phytotoxic symptoms under abiotic stress in vascular plants^10–12^ and green algae^4,13^, there is limited information regarding the effects of toxic metals on the levels of endogenous phytohormones^14–16^.

Under heavy metal stress, lower levels of free forms of cytokinins and more conjugates with less biological activity were observed in wheat seedlings^17^, in the shoots of *Juniperus* communis^18^ and *Deschampsia cespitosa*^19^. Cadmium (Cd) also modifies auxin homeostasis in *Arabidopsis thaliana*^20,21^ and *Oryza sativa*^22^ resulting into altered cell differentiation and inhibition of plant growth. An excess of copper (Cu) ions can affect auxin levels in primary root apices and cotyledons of *A. thaliana*^23^. Moreover, metal-induced inhibitory effects on vascular plant and algal growth were reported to be concomitant with an increase in endogenous ABA levels^3,19,24^. Thus, it is supposed that Pb may possess the effect on the variation of different forms of endogenous phytohormones in *A. obliquus* and it is quite possible that exogenous application of auxins and cytokinins may complement endogenous phytohormone function and restore phytohormone homeostasis disturbed by toxic metal.

Treatment with phytohormones gives promising results for vascular plants^12,15,16^ and algae^13,25^ under abiotic stress. Exogenous auxins and cytokinins significantly alleviated Pb toxicity in *A. obliquus* cells by reducing oxidative stress through the activation of enzymatic and non-enzymatic antioxidant defense systems^4^. However, little information is available about the way the phytohormones influence the detoxification mechanisms involved in metal sequestration. In plants, the most common metal-binding peptides include glutathione (GSH) and phytochelatins (PCs) with a general structure (*γ*-Glu-Cys)_*n*_-Gly, where *n* = 2–11. They are synthesized from GSH in the presence of heavy metals in the reaction catalyzed by the enzyme phytochelatin synthase (PCS)^26,27^. Detoxification of heavy metals occurs through a complex formation of PCs with metals through thiol coordination of the cysteine (Cys) present in the PC structure. Protective mechanisms involving PCs and their precursors were induced in algal cells in response to metals aimed at ameliorating eventual toxicity^3,28–30^. To our knowledge, no attempt has been made to test whether exogenous auxins and cytokinins could affect the pattern of the non-protein thiol pool, such as PCs, their precursors (Cys; *γ*-glutamylcysteine, *γ-*Glu-Cys; GSH) and the activity of PCS in green algae. In particular, cytokinins modulate the expression of metallothioneins in Cu-stressed tobacco plants^31^, can increase the level of GSH in maize treated with herbicide^32^, regulate the synthesis of PCs in *Alyssum murale* exposed to nickel (Ni)^33^ and in *A. thaliana* treated with arsenic (As)^34^.

The study aimed to assess the effect of exogenous application of structurally different auxins (IAA, IBA, PAA) and cytokinins (*t*Z, Kin, DPU) on *A. obliquus’s* ability to counteract Pb phytotoxicity. Different parameters were studied: relative changes in low-weight metabolite, the intracellular concentration of non-protein thiols (Cys, *γ*-Glu-Cys, GSH, PCs), the activity of PCS, and the profile of endogenous phytohormones. Currently, no studies are available regarding the application of plant growth regulators on green microalgae, in particular on the role of different auxins and cytokinins in Pb detoxification and homeostasis of endogenous phytohormones. Results could benefit the technology of Pb phytoextraction and add new knowledge to the mechanism of heavy metal tolerance, including PC synthesis and low molecular weight metabolites accumulation, which all are critical for Pb detoxification and sequestration. The hypothesis that auxins and cytokinins are essential components of strategies to the adaptation of algal cells to unfavorable conditions of the aquatic environment is taken into consideration.

## Results

### Endogenous phytohormone profile

The treatment with Pb caused a decrease in the intracellular levels of both IAA (by 33%) and PAA (by 45%) in *A. obliquus* cells as compared with the control (Table 1). The application of exogenous auxins to cultures stressed with Pb increased the endogenous IAA level. Thus IAA content was stimulated by 20% in cells treated with IAA + Pb by 11% in response to IBA + Pb and by 9% in cells exposed to PAA + Pb. The level of endogenous PAA was restored to the level of control cultures in response to exogenous auxins applied with Pb.

**Table 1:** The effect of exogenous auxins (IAA, IBA, and PAA) and cytokinins (*t*Z, Kin, and DPU) on the level of different forms of endogenous auxins and cytokinins in *A. obliquus* cells exposed to lead (Pb) on the 5th day of cultivation in relation to control (C).

Phytohormone content (amol cell^−1^)	Treatment							
	C	Pb	IAA+Pb	IBA+Pb	PAA+Pb	*t*Z+Pb	Kin+Pb	DPU+Pb
Indole-3-acetic acid (IAA)	176.35±4.12^e^	117.57±2.59^f^	211.65±1.16^b^	195.90±3.17^d^	192.60±3.78^d^	220.16±4.32^a^	202.06±3.04^c^	190.39±2.60^d^
%	0	−33.3	20.0	11.1	9.2	24.8	14.6	8.0
Phenylacetic acid (PAA)	375.20±2.47^c^	207.36±3.14^d^	378.16±3.53^c^	382.07±2.99^c^	407.75±2.36^b^	421.06±5.11^a^	395.53±2.57^c^	390.44±4.11^c^
%	0	−44.7	nd	nd	8.7	12.2	5.4	nd
**Σauxins**	**551.55±6.59**^**c**^	**324.93±5.73**^**d**^	**589.81±4.69**^**c**^	**577.97±6.16**^**c**^	**600.35±6.14**^**b**^	**641.22±9.43**^**a**^	**597.59±5.61**^**b**^	**580.83±6.71**^**c**^
**%**	**0**	−**41.1**	**6.9**	**nd**	**8.8**	**16.3**	**8.3**	**5.3**
*trans*-Zeatin (*t*Z)	15.34±1.01^c^	8.55±0.99^d^	15.87±2.03^c^	15.19±0.587^c^	14.87±1.36^c^	23.21±0.78^a^	19.24±1.01^b^	18.98±1.55^b^
%	0	−44.3	nd	nd	nd	51.3	25.4	23.7
*trans*-Zeatin-Riboside *(t*ZR)	0.48±0.07^c^	0.35±0.04^d^	0.52±0.03^b^	0.49±0.05^c^	0.41±0.78^c^	0.67±0.18^a^	0.59±0.03^b^	0.55±0.12^b^
%	0	−27.1	8.3	nd	−14.6	39.6	22.9	14.6
*trans*-Zeatin-9-Glucoside (*t*Z9G)	8.34±1.06^c^	10.65±1.11^a^	9.56±0.72^b^	8.45±1.26^c^	9.43±1.52^b^	9.04±0.89^b^	9.43±0.59^b^	8.92±0.63^c^
%	0	27.7	14.6	nd	13.1	8.4	13.1	7.0
*trans*-Zeatin-7-Glucoside (*t*Z7G)	0.61±0.12^d^	0.99±0.07^b^	0.53±0.23^e^	0.75±0.09^c^	0.76±0.17^c^	1.02±0.15^a^	0.91±0.05^b^	0.60±0.09^d^
%	0	62.3	−13.1	23.0	24.6	67.2	49.2	nd
*trans*-Zeatin-*O*-Glucoside (*t*ZOG)	28.46±1.06^b^	17.55±1.06^d^	26.9±1.06^c^	29.11±1.06^b^	32.71±1.06^a^	33.33±1.06^a^	29.78±1.06^b^	24.08±1.06^c^
%	0	−38.3	−5.5	nd	14.9	17.1	nd	−15.4
*trans*-Zeatin-*O*-Glucoside Riboside (*t*ZROG)	0.21±0.07^c^	0.58±0.07^a^	0.23±0.07^c^	0.24±0.07^c^	0.32±0.07^b^	0.36±0.07^b^	0.38±0.07^b^	0.19±0.07^d^
%	0	176.2	9.5	14.3	52.4	71.4	81.0	−9.5
**Σ*t*Z-types**	**53.44±3.39**^**c**^	**38.67±3.34**^**d**^	**53.61±4.14**^**c**^	**54.23±3.12**^**c**^	**58.5±4.96**^**b**^	**67.86±3.13**^**a**^	**60.33±2.81**^**b**^	**53.32±3.52**^**c**^
**%**	**0**	−**27.6**	**nd**	**nd**	**9.5**	**27.0**	**12.9**	**nd**
*cis*-Zeatin (*c*Z)	358.95±3.39^c^	276.21±3.39^d^	370.55±3.39^b^	352.18±3.39^c^	350.73±3.39^c^	397.44±3.39^a^	371.85±3.39^b^	368.16±3.39^b^
%	0	−23.1	nd	nd	nd	10.7	nd	nd
*cis*-Zeatin-*O*-Glucoside (*c*ZOG)	12.36±0.14^e^	20.37±0.91^a^	13.76±0.56^d^	14.71±0.52^c^	14.88±0.41^c^	15.63±0.13^b^	13.59±0.27^d^	10.27±0.91^f^
%	0	64.8	11.3	19.0	20.4	26.5	10.0	−16.9
*cis*-Zeatin-Riboside (*c*ZR)	108.13±3.53^b^	59.88±4.11^d^	119.05±2.07^a^	98.03±1.98^c^	95.71±1.55^c^	115.9±4.18^a^	95.78±3.07^c^	91.85±3.18^c^
%	0	−44.6	10.1	−9.3	−11.5	7.2	−11.4	−15.1
*cis*-Zeatin-*O*-Glucoside Riboside (*c*ZROG)	1.94±0.13^d^	3.67±0.09^b^	2.11±0.14^c^	3.52±0.04^b^	2.14±0.12^c^	4.18±0.02^a^	2.37±0.06^c^	1.69±0.13^d^
%	0	89.2	8.8	81.4	10.3	115.5	22.2	−12.9
***ΣcZ*-types**	**479.44±7.19**^**c**^	**360.13±8.50**^**d**^	**505.47±6.16**^**b**^	**468.44±5.93**^**c**^	**463.46±5.47**^**c**^	**533.15±7.72**^**a**^	**483.59±6.79**^**c**^	**471.97±7.61**^**c**^
**%**	**0**	−**24.9**	**5.4**	**nd**	**nd**	**11.2**	**nd**	**nd**
Dihydrozeatin (DHZ)	21.44±1.16^b^	10.20±0.99^c^	20.47±1.23^b^	19.83±1.24^b^	19.92±0.97^b^	25.76±1.07^a^	24.30±1.10^a^	20.21±0.75^b^
%	0	−52.4	nd	−7.5	−7.1	20.1	13.3	−5.7
Dihydrozeatin Riboside (DHZR)	28.51±3.04^c^	14.38±1.11^d^	27.42±1.93^c^	25.89±2.13^c^	26.08±2.08^c^	30.70±1.17^b^	32.06±1.66^a^	30.10±1.67^b^
%	0	−49.6	nd	−9.2	−8.5	7.7	12.5	5.6
Dihydrozeatin-*O*-Glucoside (DHZOG)	5.22±0.09^d^	8.56±0.16^a^	6.18±0.17^c^	7.02±0.13^b^	7.22±0.24^b^	5.18±0.08^d^	5.55±0.21^d^	6.28±0.11^c^
%	0	64.0	18.4	34.5	38.3	nd	6.3	20.3
Dihydrozeatin-9-Glucoside (DHZ9G)	0.74±0.06^c^	1.24±0.05^a^	0.80±0.11^b^	1.26±0.25^a^	0.95±0.17^b^	0.78±0.10^c^	0.83±0.09^b^	0.77±0.04^c^
%	0	67.6	8.1	70.3	28.4	5.4	12.2	nd
Dihydrozeatin-7-Glucoside (DHZ7G)	0.50±0.04^d^	1.31±0.15^a^	0.94±0.08^b^	0.73±0.03^c^	0.75±0.07^c^	0.48±0.03^d^	0.53±0.04^d^	0.72±0.10^c^
%	0	162.0	88.0	46.0	50.0	nd	6.0	44.0
**ΣDHZ-types**	**56.41±4.39**^**b**^	**35.69±2.46**^**c**^	**55.81±3.52**^**b**^	**54.73±3.78**^**b**^	**54.92±3.53**^**b**^	**62.90±2.45**^**a**^	**63.27±3.10**^**a**^	**58.08±2.67**^**b**^
**%**	**0**	−**36.7**	**nd**	**nd**	**nd**	**11.5**	**12.2**	**nd**
*N*^*6*^-Isopentenyladenine (iP)	43.65±2.12^c^	25.80±1.09^d^	46.12±2.02^c^	44.03±2.16^c^	42.87±1.48^c^	56.07±3.60^a^	48.29±1.88^c^	51.19±1.63^b^
%	0	−40.9	5.7	nd	nd	28.5	10.6	17.3
*N*^6^-Isopentenyladenosine (iPR)	55.88±1.65^b^	30.10±1.54^d^	35.94±1.66^c^	59.35±2.07^b^	56.70±3.12^b^	70.81±2.04^a^	58.41±2.13^b^	56.26±1.11^b^
%	0	−46.1	−35.7	6.2	nd	26.7	nd	nd
*N*^*6*^-Isopentenyladenine-7-Glucoside (iP7G)	1.34±0.15^d^	3.77±0.22^a^	2.21±0.07^c^	2.09±0.07^c^	3.01±0.16^b^	1.33±0.06^d^	1.55±0.14^d^	1.50±0.11^d^
%	0	181.3	64.9	56.0	124.6	nd	15.7	11.9
**ΣiP-types**	**100.87±3.92**^**b**^	**59.67±2.85**^**d**^	**84.27±3.75**^**c**^	**105.47±4.30**^**b**^	**102.58±4.76**^**b**^	**128.21±5.70**^**a**^	**108.25±4.15**^**b**^	**108.95±2.85**^**b**^
%	**0**	−**40.8**	−**16.5**	**nd**	**nd**	**27.1**	**7.3**	**8.0**
**ΣTotal CKs**	**690.12±18.89**^**c**^	**494.16±17.15**^**d**^	**699.16±17.57**^**c**^	**682.87±17.13**^**c**^	**679.46±18.72**^**c**^	**791.89±19.00**^**a**^	**715.44±16.85**^**b**^	**692.32±16.65**^**c**^
**%**	**0**	−**41.1**	**6.9**	**nd**	**8.8**	**16.3**	**8.3**	**5.3**

Treatment with at least one letter the same are not significantly different according to Tukey’s Post-hoc test. % indicates the percentage increase or decrease (negative numbers) in phytohormone level compared to the control culture; nd means no differences between the control and algal cultures treated with Pb and/or phytohormones.

Cytokinins were characterized by higher stimulating effect on endogenous auxin level in cells growing under Pb stress. The combined treatment of algal cells with the mixture of *t*Z + Pb stimulated IAA and PAA contents by 25% and 12%, respectively. Kin applied together with heavy metal induced 15% and 5% increase in IAA and PAA contents, respectively. Synthetic cytokinin, DPU increased IAA level by 8%. However, this phytohormone did not exert a statistically significant influence on the endogenous PAA level in algal cells subjected to Pb stress.

The active cytokinins, including *t*Z, iP, DHZ, and *c*Z were detected in *A. obliquus* (Table 1). Pb stress was associated with strong suppression of active cytokinin content by 27% in comparison with control. Cytokinin ribosides (the transport forms) significantly decreased by 36% upon Pb treatment. The levels of active cytokinins may be diminished by their glucosylation, either at the purine ring (*N*-glucosylation) or at the side chain (*O*-glucosylation). Pb stimulated this process leading to an increase in the contents of *N*- and *O*-glucosides of cytokinins by 55% and 6%, respectively.

Exogenous auxins and cytokinins induced changes in endogenous contents of different forms of cytokinins in algal cultures exposed to heavy metal stress (Table 1). The highest increase in cytokinin bases was observed in cells exposed to *t*Z + Pb, because the levels of endogenous *t*Z, *c*Z, DHZ, and iP were stimulated by 51%, 11%, 20%, and 28%, respectively. Other phytohormones restored the homeostasis of cytokinin bases to the level observed in control. Among auxins, IAA used in combination with Pb increased the levels of cytokinin ribosides by 8% and 10% in the case of *t*ZR and *c*ZR, respectively; whereas the content iPR decreased by 36%. IBA and PAA were less active auxins in the restoration of cytokinin riboside levels. The intracellular contents of *t*ZR, *c*ZR, DHZR, and iPR were higher by 40%, 7%, 8%, and 27%, respectively, in algal cultures treated with both *t*Z and Pb. Other cytokinins, Kin and DPU, were characterized by lower activity regarding the impact on homeostasis of riboside forms of cytokinins in algal cells treated with Pb. In response to exogenous auxins and cytokinins in combination with heavy metal, the increases in the amounts of endogenous *N*-glucosides of cytokinins were noted. The application of IAA + Pb, IBA + Pb and PAA + Pb resulted in 22%, 15% and 29% rise in the content of *N*-glucosides, whereas *t*Z + Pb, Kin + Pb and DPU + Pb increased their level by 10%, 15% and 8% in comparison with the control cultures. Among *N*-glucosides, the highest increase in their accumulation was observed in the case of DHZ7G, because its level was stimulated by 88% in response to IAA and Pb. The increase in the contents of total *O*-glucosides was obtained in algal cells treated with IBA + Pb (13%), PAA + Pb (19%), *t*Z + Pb (22%) and Kin + Pb (7%). Adenine cytokinin, *t*Z in compilation with heavy metal-induced higher rise in accumulation of *t*ZOG by 17%, *t*ZROG by 71%, *c*ZOG by 26%, and *c*ZROG by 115% in relation to control. However, the levels of *N*- and *O*-glucosides were lower than in cultures treated with Pb alone.

**Figure 1:**
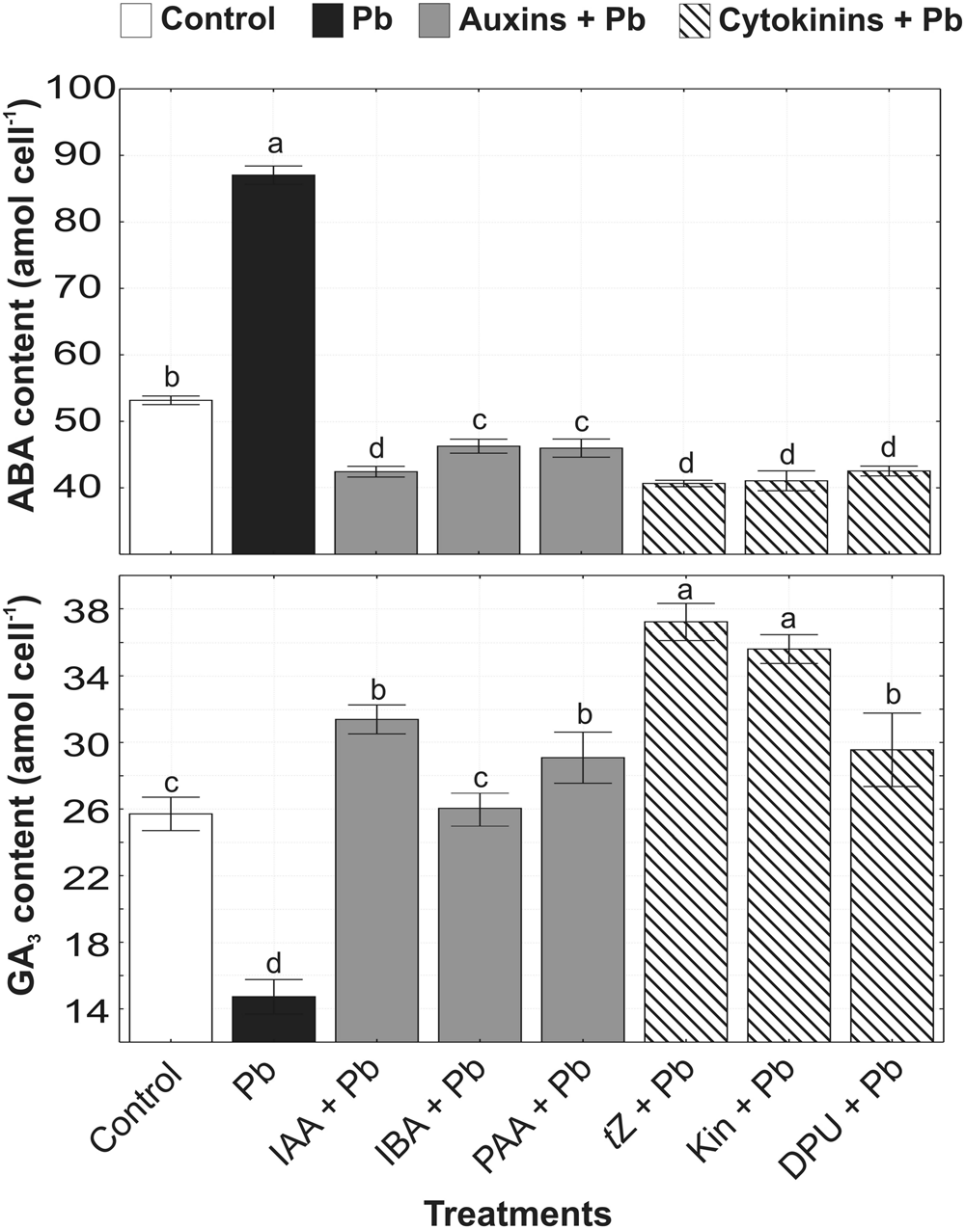
The effect of auxins (0.01 µM IAA, 0.1 µM IBA, 0.1 µM PAA) and cytokinins (0.01 µM *t*Z, 0.1 µM Kin, 1 µM DPU) on abscisic acid (ABA) and gibberellin (GA3) content in *A. obliquus* exposed to 100 µM Pb in relation to control on the 5th day of culture. Data are the means of four independent experiments ± SD. Treatment with at least one letter the same are not significantly different according to Tukey’s Post-hoc test.

The exposure of *A. obliquus* cells to Pb stress increased ABA level by 111% in relation to control (Fig. 1). Application of auxins and cytokinins induced a decrease in ABA level in algal cultures exposed to Pb. The highest reduction in endogenous ABA content by 22% and 25% was observed in cultures treated with IAA + Pb and *t*Z + Pb, respectively. Other auxins (IBA, PAA) and cytokinins (Kin, DPU) less effectively decreased ABA level in algal cells growing in the presence of heavy metal.

The treatment of algal cells with Pb induced a significant reduction in GA3 accumulation by 41% in relation to control (Fig. 1). All studied phytohormones stimulated gibberellin (GA3) content in algal cells growing in the presence of heavy metal. For example, IAA + Pb (among auxins) and *t*Z + Pb (among cytokinins) increased GA3 level by 16% and 38%, respectively, in comparison with the control cultures.

### Principal component analysis (PCA) of phytohormone profile in algal cells in response to auxins and cytokinins under Pb stress

**Figure 2:**
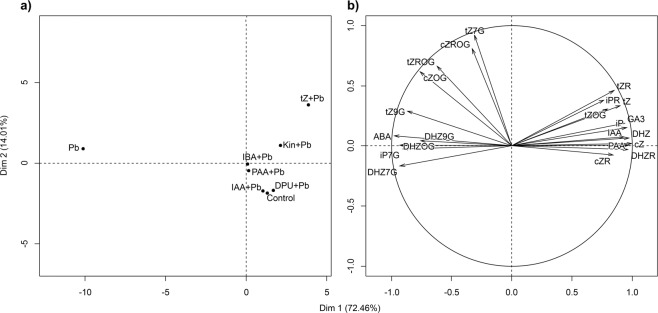
Phytohormone profile of *Acutodesmus obliquus*. (**a**) PCA score plot and (**b**) PCA loadings plot for the first two factors (Dim 1–2) that together explain about 86.47% of variance.

Positive scores for the first principal component (Dim 1) indicate higher values of *t*ZR, iPR, *t*Z, *t*ZOG, GA_3_, iP, IAA, DHZ, PAA, *c*Z, *c*ZR, and DHZR, while negative show lower values of *t*Z7G, *c*ZROG, *t*ZROG, *c*ZOG, *t*Z9G, DHZ9G, ABA, DHZOG, iP7G and DHZ7G, compared to mean values for all studied algal treatments (Fig. 2). All the positive scores are grouped together, thus highly correlated. Furthermore, positive scores for the second principal component (Dim 2) indicate higher values of *t*Z7G, *c*ZROG, *t*ZROG, and *c*ZOG, compared to mean values for all the studied algal treatments. The phytohormones show a very good quality of representation on the created model. PCA clustered algal treatment with IBA + Pb and PAA + Pb. Their phytohormone profiles are almost equal to the overall mean for each of the phytohormones. DPU + Pb, IBA + Pb, and control were also grouped, suggesting that the two combined treatments do not show significant effect compared to the control. Those two groups of treatments differ in the values of *t*Z7G, *c*ZOG, DHZOG, DHZR, and *c*Z. The treatment with Kin + Pb shows the highest value of DHZR and GA_3_.The treatment with Pb alone is the only one characterized by negative loadings (low levels) in the first dimension and the highest Euclidean distance with the origin, suggesting it had the greatest impact on the phytohormones profile in *A. obliquus* cells. However, the treatment with *t*Z + Pb showed the highest positive loadings (high levels). Therefore, it is characterized by the highest levels of, e.g., *t*Z, *t*ZR, *t*Z7G, *c*ZROG, *c*ZR, and iPR, and lowest of e.g., ABA, DHZOG, and DGZ9G.

### Intracellular metabolite profile

Analysis of metabolite profile (Table 2) of *A. obliquus* cells indicated that among primary amino acids, the contents of glycine (Gly), glutamate (Glu), and homocysteine showed a significant reduction in response to Pb stress.

**Table 2:** Metabolites which levels significantly changed in *A. obliquus* cells exposed to lead (Pb) and auxins (IAA, IBA, and PAA) as well as cytokinins (*t*Z, Kin, and DPU) applied with Pb on the 5th day of cultivation.

Metabolite	Treatment						
	Pb	IAA + Pb	IBA + Pb	PAA + Pb	*t*Z + Pb	Kin + Pb	DPU + Pb
Alanine	18.3	nd	−5.5	nd	−5.7	nd	5.6
Glutamate	−5.9	−21.9	−16.7	−7.8	−31.5	−27.9	−19.2
Glutamine	32.5	15.4	10.2	6.6	25.7	20.4	12.5
Glycine	−39.7	−88.9	−75.1	−62.4	−80.5	−74.1	−55.5
Homocysteine	−37.2	−68.1	−62.6	−59.3	−70.2	−65.3	−61.2
Isoleucine	21.8	−15.6	−10.4	nd	nd	−15.8	−7.3
Leucine	28.5	nd	nd	nd	−8.5	−18.3	−8.2
Phenylalanine	44.0	−10.3	nd	nd	−12.6	nd	7.3
Proline	21.7	59.3	50.1	48.9	78.2	65.7	48.2
Threonine	22.4	nd	nd	nd	−6.0	nd	7.4
Tyrosine	36.6	−17.6	−7.5	nd	−19.1	−9.5	nd
Valine	29.5	nd	nd	nd	−10.5	nd	nd
Inositol	18.9	35.6	36.2	24.7	45.5	35.6	32.4
Citric acid	35.7	55.6	51.0	49.0	66.5	60.8	42.5
Fumaric acid	8.1	nd	5.5	nd	21.2	20.4	21.0
Gluconic acid	6.3	10.5	11.9	nd	nd	nd	nd
Malic acid	31.0	39.6	33.4	34.0	47.5	40.0	36.8
Arachidonic acid	30.1	55.3	50.7	38.5	68.1	60.2	41.7
Hexadecanoic acid	21.5	58.9	42.1	38.6	67.5	55.8	60.2
Heptadecanoic acid	20.6	38.1	30.3	29.0	43.4	37.3	21.6
Linoleic acid	34.8	55.5	54.6	50.5	68.2	55.0	49.2
Linolenic acid	36.5	49.8	47.1	46.2	66.1	60.8	53.4
Octadecanoic acid	15.4	42.3	40.8	31.6	49.7	38.4	35.9
Oleic acid	24.0	36.5	35.2	31.3	58.0	50.1	39.6

The numbers indicate the percentage increase or decrease (negative numbers) in metabolite level compared to the control culture; nd means no differences between the control and algal cultures treated with Pb and/or phytohormones.

The application of auxins or cytokinins together with heavy metal has deepened this trend because their contents decreased further. The levels of aromatic (phenylalanine, tyrosine), branched-chain amino acids (valine, leucine, isoleucine), and other amino acids (alanine, glutamine, proline) increased in microalga growing under the influence of Pb. Algal cells treated with auxins or cytokinins in combination with heavy metal were characterized by a decrease in the level of these amino acids. A significant rise in the content of proline and organic acids (citrate, malate, fumarate, gluconate) was observed in algal cells in response to auxins or cytokinins applied together with Pb. Auxins or cytokinins induced the highest rise in the contents of fatty acids such as hexadecanoic, heptadecanoic, linoleic, linolenic, octadecanoic, and oleic acids in *A. obliquus* cells growing in the presence of Pb. Generally, IAA among auxins and *t*Z among cytokinins induced the most significant changes in algal metabolome in stress conditions.

### Phytochelatin precursors

**Figure 3:**
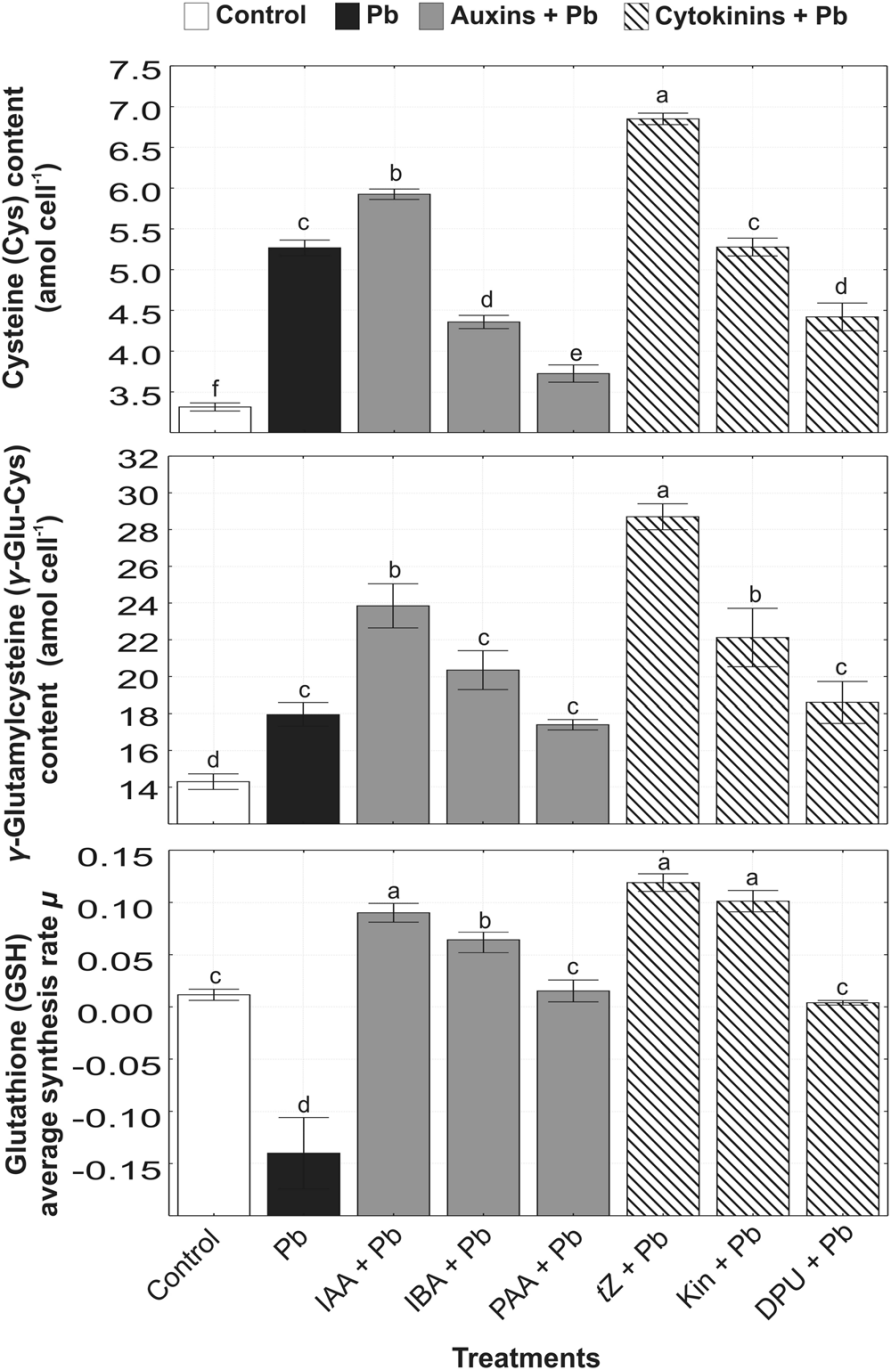
The effect of auxins (0.01 µM IAA, 0.1 µM IBA, 0.1 µM PAA) and cytokinins (0.01 µM *t*Z, 0.1 µM Kin, 1 µM DPU) on the content of cysteine and *γ*-glutamylcysteine and glutathione synthesis rate in *A. obliquus* exposed to 100 µM Pb in relation to control on the 5th day of culture. Data are the means of four independent experiments ± SD. Treatment with at least one letter the same are not significantly different according to Tukey’s Post-hoc test.

The accumulations of thiol-containing compounds (Cys, *γ*-Glu-Cys) compared to the controlwere observed in all tested variants (Fig. 3), although their levels were higher in algal cultures treated with auxins and cytokinins under Pb stress. The most active auxin, IAA, in combination with Pb stimulated the amounts of Cys and *γ*-Glu-Cys by 79% and 67%, respectively. Among cytokinins, *t*Z the most effectively increased Cys and *γ*-Glu-Cys contents by 107% and 101%, respectively, in *A. obliquus* cells under Pb stress. Other auxins (IBA, PAA) and cytokinins (Kin, DPU) possessed a weaker stimulatory impact on the synthesis of thiol compounds in cells subjected to heavy metal stress. Moreover, the presence of Pb in algal cultures inhibited the average synthesis rate of GSH (Fig. 3). Application of exogenous auxins and cytokinins stimulated the synthesis of this peptide in *A. obliquus* exposed to Pb stress. The highest average synthesis rate of GSH was noted in algal cultures treated with IAA + Pb (0.09) and *t*Z + Pb (0.119).

### Phytochelatin synthase activity

**Figure 4:**
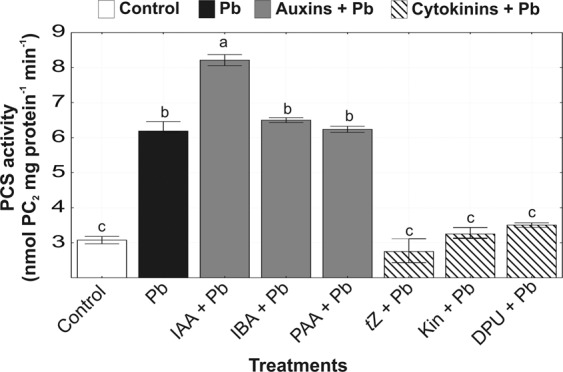
The effect of auxins (0.01 µM IAA, 0.1 µM IBA, 0.1 µM PAA) and cytokinins (0.01 µM *t*Z, 0.1 µM Kin, 1 µM DPU) phytochelatin synthase (PCS) activity in *A. obliquus* exposed to 100 µM Pb in relation to control on the 5th day of culture. Data are the means of four independent experiments ± SD. Treatment with at least one letter the same are not significantly different according to Tukey’s Post-hoc test.

Pb doubled the activity of PCS in comparison with the control (Fig. 4). Coapplication of auxins or cytokinins with heavy metal regulated the activity of an enzyme involved in PC synthesis in relation to cultures treated with Pb alone. The highest rise in activity of PCS (by 167%) occurred in *A. obliquus* cells treated with IAA + Pb. Other auxins in combination with heavy metals accelerated PCS activity by 111% (IBA + Pb) and 103% (PAA + Pb) as compared to control. Cytokinins were characterized by an opposite impact on PCS in relation to auxins. Coapplication of *t*Z with Pb inhibited PCS activity by 11%. On the other hand, Kin + Pb did not exert a statistically significant effect on this enzyme, whereas the DPU + Pb enhanced (by 13%) reaction catalyzed by PCS in relation to control.

### Phytochelatin content

**Figure 5:**
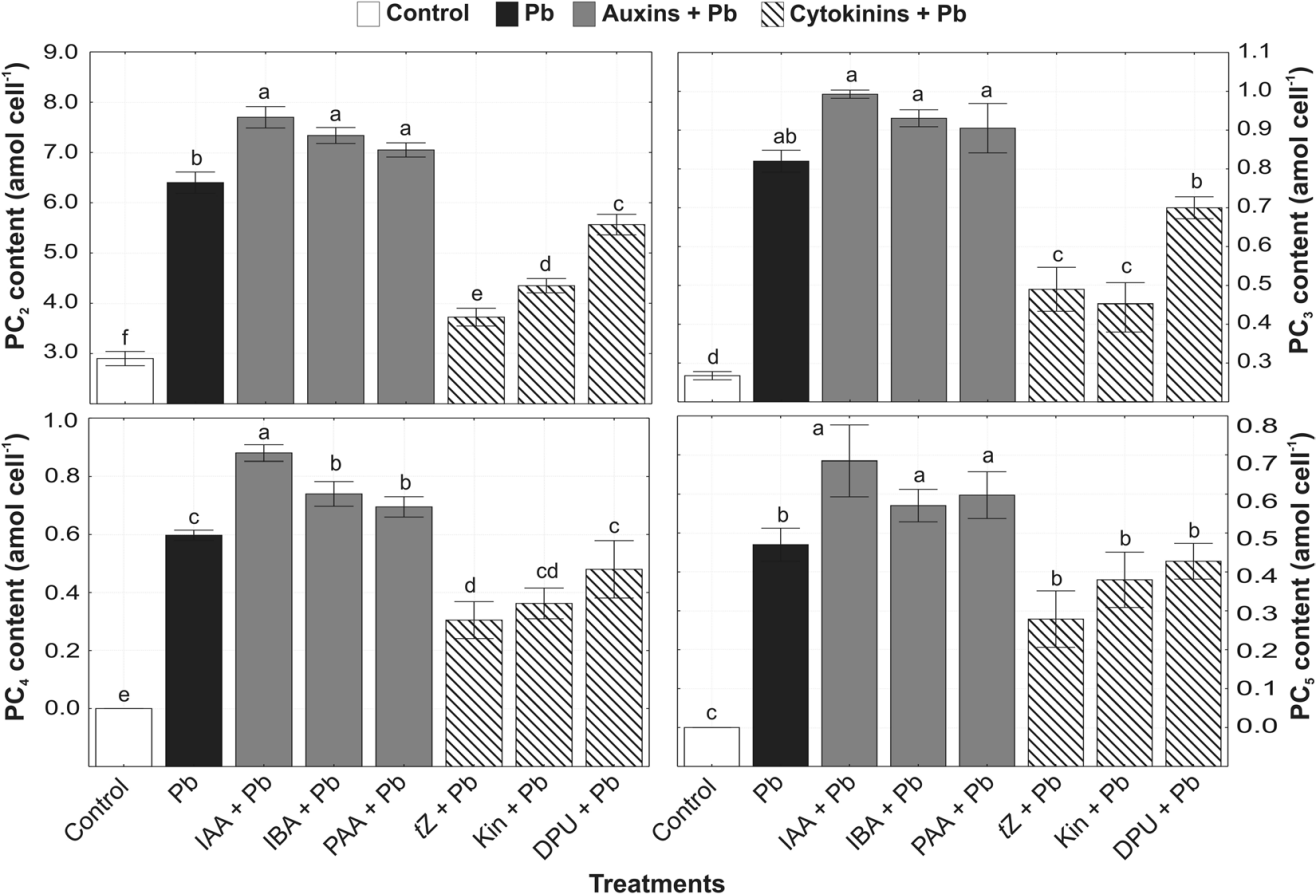
The effect of auxins (0.01 µM IAA, 0.1 µM IBA, 0.1 µM PAA) and cytokinins (0.01 µM *t*Z, 0.1 µM Kin, 1 µM DPU) on the content of phytochelatins in *A. obliquus* exposed to 100 µM Pb in relation to control on the 5th day of culture. Data are the means of four independent experiments ± SD. Treatment with at least one letter the same are not significantly different according to Tukey’s Post-hoc test.

The addition of Pb to the medium induced the synthesis of PCs (6.40 amol cell^−1^ PC_2_, 0.82 amol cell^−1^ PC_3_, 0.60 amol cell^−1^ PC_4_, 0.47 amol cell^−1^ PC_5_) in *A. obliquus* cells (Fig. 5). The application of auxins to microalgal cultures growing in the presence of Pb increased the amounts of GSH-related peptides more effectively compared to heavy metal alone. The highest increases in the contents of PCs (7.70 amol cell^−1^ PC_2_, 0.99 amol cell^−1^ PC_3_, 0.88 amol cell^−1^ PC_4_, 0.69 amol cell^−1^ PC_5_), were observed in response to the mixture of IAA with Pb as compared to control. Other auxins, IBA and PAA were characterized by lower, but statistically not significant in comparison with IAA, stimulatory effect on synthesis of non-protein thiols in Pb-treated algal cells. Among cytokinins, *t*Z displayed the strongest inhibitory influence on the level of these chelating peptides except to PC_5_. Exogenously added Kin and DPU were observed to be less effective in the inhibition of PC synthesis in microalga growing under Pb stress.

**Figure 6:**
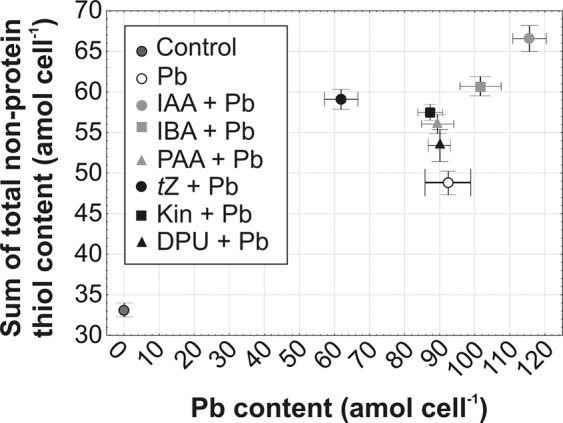
The content of non-protein thiol compounds calculated as (∑SHtot = GSH + [2 × PC_2_ + 3 × PC_3_ + 4 × PC_4_ + 5 × PC_5_] as a function of intracellular Pb content on 5th day of cultivation in response to auxins (0.01 µM IAA, 0.1 µM IBA, 0.1 µM PAA) and cytokinins (0.01 µM *t*Z, 0.1 µM Kin, 1 µM DPU). Data are the means of four independent experiments ± SD.

The large increases in the levels in total thiols [∑SH_tot_] were observed in algal cultures treated with auxins which were positively correlated with higher Pb contents (Fig. 6). For example, IAA stimulated Pb content in algal cells to 115.46 amol cell^−1^ and therefore induced the accumulation of the sum of total thiols to 66.57 amol cell^−1^. On the other hand, cytokinins reduced Pb content in *A. obliquus* cells, and therefore the level of thiol compounds was lower in relation to cultures treated with auxins in combination with heavy metal. The most active cytokinin *t*Z inhibited Pb level to 61.92 amol cell^−1^ and decreased the sum of total thiols to 59.03 amol cell^−1^.

## Discussion

Pb possesses a toxic effect on green microalga *A. obliquus*, reducing the cell number in the cultures. Algal growth suppression may be associated with the down-regulation of auxins, cytokinins, and gibberellins involved in the induction of cell division with a simultaneous increase in ABA content in microalga^3^. Thus, alternative ways and potential strategies to counteract heavy metal toxicity could be the application of exogenous auxins (IAA, IBA, PAA) and cytokinins (*t*Z, Kin, DPU) to improve algal growth and viability.

The results of principal component analysis (PCA) indicated that Pb and exogenous auxins and cytokinins applied with heavy metal possessed strong influence on the levels of endogenous phytohormones in *A. obliquus*. The intracellular amounts of auxins (IAA, PAA) decreased following Pb stress contributing to growth inhibition as previously observed in *A. obliquus* cultures^3,4,9^. The effect of metals on auxin content and the related alterations in growth and metabolism is extensively explored in vascular plants^21^. As stress causes toxicity by changing the levels of auxins in *B. juncea*^35^. Cu can affect auxin levels in primary root apices and cotyledons^23^, and Al alters both auxin accumulation and its distribution in *A. thaliana*^36^. The results reported here show that exogenous auxins (IAA, IBA, and PAA) and cytokinins (*t*Z, Kin, and DPU) reverse the inhibitory effect of Pb on *A. obliquus* through the stimulation of the level of intracellular auxins which are the key components of the cell cycle and signal transduction pathways involved in cell proliferation and stress tolerance.

Cytokinins are essential plant hormones responsible for cell cycle progression in vascular plants and algae^5,7,8^. When Pb was added to the nutrient medium, there was a substantial unbalance between individual forms of cytokinins. The decrease in the levels of free bases of cytokinins in *A. obliquus* cells was observed in Pb-treated cells. A similar profile was exhibited by their precursors - cytokinin ribosides. Cytokinin deactivation by *N*- and *O*-glucosylation was increased in response to Pb in order to contribute to the down-regulation of the active pool of cytokinins. Similar results were observed in the shoots of *J. communis* growing in the presence of different metals^18^, wheat seedlings^17^ and *D. cespitosa*^19^ treated with Cd, *A. thaliana* leaves exposed to ZnO nanoparticle^37^ and hyperaccumulator *Pteris cretica* var*. nervosa*^16^ subjected to As stress.

The use of exogenous auxins and cytokinins ameliorated the adverse effect of Pb on the pool of endogenous cytokinins, which may indicate the tendency to maintain cytokinin homeostasis in *A. obliquus* and minimize metal toxicity on algal growth^4^. The contents of free bases, ribosides, and *O*-glucosides of cytokinins were stimulated or reversed to the level observed in control. The levels of *N*-glucosides were still high in response to auxins and cytokinins in algal cultures growing in the presence of Pb, however significantly lower in comparison with the cultures treated with toxic metal alone. The most prominent changes were observed in the case of *t*Z + Pb and IAA + Pb treatment. The increase in contents of endogenous free bases of cytokinins (*t*Z, *c*Z, iP, DHZ) and their corresponding ribosides to the amounts observed in control after auxin and cytokinin exposures in heavy metal stress conditions can be interpreted as a detoxification mechanism to counteract the metal stress. Elevated levels of cytokinins are advantageous for the survival of toxic metals, as was observed in tobacco leaves^31^. Steady cytokinin levels in algal cells treated with exogenous auxins and cytokinins in stress conditions may be necessary for the stabilization of the photosynthetic machinery and stimulation of cell cycle proliferation in *A. obliquus* cultures exposed to Pb^4^. Summarizing, cytokinins and auxins probably play a synergistic role in *A. obliquu*s response to metal stress, but the specific mechanism that modulates this crosstalk is unknown.

A substantial increase in levels of ABA, which down-regulates plant growth and cell division, was detected in Pb-treated in *A. obliquus* cells, highlighting that ABA was a hormone affected explicitly by the metal. A rapidly elevated ABA level under Pb stress conditions suggests that this phytohormone also plays a crucial role as a modulator of algal stress symptoms and responses. These results are in agreement with the data indicating that exposure to heavy metals (e.g., Cd and Zn) induces expression of ABA biosynthetic genes and in turn, increases the endogenous levels of ABA which activates the specific signaling pathways and modulates gene expression involved in stress response in vascular plants^37–39^. Exogenous application of cytokinins (*t*Z, Kin, DPU) and auxins (IAA, IBA, PAA) reduced ABA content in *A. obliquus* cultures growing in the presence of Pb. Thus, an ameliorative effect of phytohormones on algal growth and tolerance as previously has been observed^3,4^ upon metal stress may be connected with a reduction in ABA level.

The treatment of green alga *A. obliquus* with Pb induced a significant reduction in the endogenous level of GA_3_, a phytohormone which strongly affects the rate of cell division and plant growth. All studied phytohormones stimulated gibberellin content in algal cells leading to the enhanced tolerance of algal cells to Pb stress. Gibberellin-mediated alleviation of stress was confirmed in vascular plants. For example, GA_3_ was reported to alleviate heavy metal toxicity by reducing toxic metal uptake and lipid peroxidation^40^, by protecting photosynthesis and regulating the ionic distribution and hormonal homeostasis in *Cicer arietinum*^41^ and wheat plants^42^.

This paper is, to our knowledge, the first report describing the effect of Pb on different plant hormone groups simultaneously in microalga and the impact of exogenously applied auxins and cytokinins during heavy metal stress on phytohormone homeostasis. Moreover, almost no information is available about the hierarchic relationship between the effect of metal on the level of plant hormones and PCs as well as their precursors in the context of microalgae.

To identify the molecular mechanism that underlies auxin- and cytokinin-mediated tolerance to Pb stress, GC-MS screening of low weight molecular metabolites was analyzed in *A. obliquus*. Co-application of auxins or cytokinins with Pb increased the levels of free fatty acids. One possible reason for the increase in the contents of fatty acids is that, as shown in previous studies^4,13^ the application of auxins and cytokinins to algal cultures prevents the formation of reactive oxygen species which can attack fatty acids leading to their oxidative degradation triggered by the presence of toxic Pb.

The exposure of *A. obliquus* to auxins and cytokinins in combination with heavy metal resulted in a significant accumulation of fumarate, gluconate, citrate, and malate, suggesting that these organic acids may play a role in metal detoxification in green alga. Organic acids can form metal-organic complexes and sequestrate them in the vacuole. Chelation of toxic metal ions by low molecular weight organic acids is an effective mechanism for the metal defense system^30,43^. Therefore, organic acid accumulation may be correlated with the metal resistance of *A. obliquus* cultures. The roles of organic acids in heavy metal chelation and tolerance were also demonstrated in other algal species. For example, microalga *Coccomyxa subellipsoidea* treated with Cd and nitric oxide (NO) donor was characterised by the increase in malate content and Cd biosorption^43^. *Scenedesmus* sp. cultures exposed to Cd or Ni indicated higher accumulation of organic acids with properties of chelation of heavy metals^30^. Morover, the presence of Cd increased the level of organic acids in non-vascular (*Taxiphyllum barbieri*) and vascular (*Ceratophyllum demersum*) aquatic plants^44^.

The increase in inositol level observed in *A. obliquus* cells treated with phytohormones may also contribute to heavy stress acclimation as it has been reported for alga *Schizochytrium* sp.^45^. A decrease in the endogenous contents of most amino acids suggested their involvement in the biosynthesis of peptides^46^ and proteins which levels were stimulated in *A. obliquus* after coapplication of auxins or cytokinins with Pb^4^. A metabolomic study also showed an upregulation of the GSH synthesis pathway as a result of the reduction in the content of homocysteine, glutamate (Glu), and glycine (Gly) in *A. obliquus* cells exposed to phytohormones in combination with Pb. First, *γ*-Glu-Cys is synthesized from Glu and Cys, after which Gly is added to the *C*-terminal of *γ*-Glu-Cys. For the production of Cys, sulfur is provided by homocysteine^46^. Elevated amounts were found for Gln, which plays a role in the synthesis of Glu. The obtained results suggest that auxins and cytokinins accelerated sulfur assimilation pathway, leading to the synthesis of GSH and increasing *A. obliquus* tolerance to Pb stress.

PCs are synthesized from GSH in a *γ*-glutamyl cysteinyl transpeptidation reaction catalyzed by enzyme PCS, which activity is dependent on the type and concentration of free metal ions^26,27^ and the phytohormones. Obtained results suggest that exogenous auxins are efficient signaling molecules in activating PCS, thus inducing the production of more PCs in *A. obliquus* cells growing in the presence of Pb. The increased PCS activity in *A. obliquus* cells exposed to both auxins and Pb was consistent with the highly increased concentration of PC_4_, suggesting that the polymerization of PC2 into PC_3_, PC_4_, and PC_5_ was a direct result of the higher PCS activity. On the other hand, the impact of exogenous cytokinins on PCS activity was phytohormone-dependent. Cytokinin *t*Z, characterized by the highest biological activity in algal cultures, was able to inhibit the reaction catalyzed by PCS. DPU enhanced PCS activity in *A. obliquus* during Pb stress. Thus the level of PCs was lower in relation to cultures treated with Pb alone; however, Kin did not exert any significant influence on this level.

Metal sequestration by PCs is one of the best well-known mechanisms of heavy metal detoxification in green microalgae due to their ability to form stable metal-PC complexes^28,29^. The phytohormone dependent intracellular content of Pb in *A. obliquus* was in parallel with the PCs production, which further revealed a relationship between PCs availability and metal accumulation. Present results confirmed previous experiments^4^ indicating that *A. obliquus* showed high capacity of Pb accumulation in response to auxins, in contrast to cultures treated with cytokinins indicating that heavy metal biosorption depends ambiguously on the phytohormones. Auxins and cytokinins regulated the content of Pb, which correlated well with the PC level in green alga. In *A. obliquus* cells, the combined auxin and metal exposure led to a higher level of total thiols, including PCs which positively correlated with enhanced Pb content in microalgal cells. However, the role of exogenous auxins in adaptation to toxic metals is still poorly understood, and only a few studies are available regarding the relationship between auxin and PCs in vascular plants. For example, exposure to Cd, Cu, and Zn, resulting in an over fivefold increase of auxin level in *A. thaliana* roots, was consistent with a higher level of PCs^21^ indicating that auxins may be involved in the stimulation of thiol peptides chelating Pb. Similarly, significant decreases in the amounts of Cys, GSH, and total PCs were observed in *O. sativa* treated with the inhibitor of auxin transport (TIBA)^47^. On the other hand, cytokinins inhibited the synthesis of PCs in *A. obliquus* cells as compared to cultures treated with metal alone, due to a lower Pb content. The negative relation between cytokinins, especially *t*Z, and PCs was also confirmed in vascular plants. For example, transgenic plants (*A. thaliana*, tobacco) with reduced levels of endogenous cytokinins showed a higher accumulation of GSH, PC_2_, PC_3_, and PC_4_ leading to higher tolerance to As stress in relation to the wild types. Transgenic *A. thaliana* overexpressed cytokinin oxidase/dehydrogenase 1 responsible for cytokinin degradation showed increased expression of genes involved in PC biosynthesis (*γ*-glutamylcysteine synthetase, GSH synthetase, and PCS)^34^. Other study showed that cytokinin treatments induced a significant increase in biomass of *A. murale* during Ni stress, whereas no significant variation in the concentration of *γ*-Glu-Cys, GSH, and total PCs was observed^33^. The present results showthat the exogenous application of cytokinins (*t*Z, Kin, and DPU) stimulated the level of active forms of endogenous cytokinins and also played a role as a negative regulator of metal detoxification machinery, a significant cause of Pb accumulation. Therefore, the ameliorative effect of cytokinins on algal cells growing in the presence of Pb is probably related to metal exclusion and antioxidative defense system, including GSH^4^ rather than stimulation of PC synthesis induced by heavy metal.

## Conclusion

Identification of auxins and cytokinins as an integral part of the Pb response in *A. obliquus* cells offers a new understanding of algal integrate phytohormone signaling pathways with adaptation to metal stress. Exogenous phytohormones ameliorate the toxic effect of heavy metal on green alga. Phytohormone homeostasis was disturbed in response to Pb, whereas the application of exogenous auxins and cytokinins alleviated this adverse effect of heavy metal. Metabolomic studies revealed that auxins and cytokinins induced changes in the level of small weight molecular metabolites involved in Pb detoxification. Amino acids, which are substrates for the synthesis of GSH, were the most influenced compounds in cultures treated with auxins and cytokinins in combination with Pb. The strong induction of synthesis and accumulation of PC_2_–PC_5_ by the improvement of PCS activity under the influence of exogenous auxins applied together with Pb was confirmed. Cytokinin-induced inhibitory effects on Pb level by algal cells were reported to be concomitant with a declined synthesis of PCs. The cytokinins act as a negative regulator of the accumulation of thiol-rich peptides, which are involved in metal chelation. The data presented in this paper can be used for developing ecological technology based on the exogenous application of phytohormones to improve tolerance under heavy metal contamination, which may contribute to the phytoremediation of polluted aquatic ecosystems.

## Material and methods

### Test organism, growth conditions and treatments

The wild-type *A. obliquus* SAG Strain No. 276–6 was purchased from the SAG Culture of Algae Collection (Germany). The growth conditions and procedures of culture synchronization are the same as previously described^3,4,9,48^. The axenic algal cultures were cultivated in Erlenmeyer flasks with Bold Basal Medium (pH 7.0; 100 mL)^49,50^ for 5 days at 25 ± 0.5 °C, in a 16: 8 (light: dark) cycle and illuminated with a light intensity of 50 µMol m^−2^ s^−1^. Using air pumps cell suspension was bubbled by atmospheric air at 1 L min^−1^ to provide necessary CO_2_. During the experiment, green alga *A. obliquus* has been grown as an unicellular form. The concentrations of analyzed compounds were selected based on previous experiments^4,9,48^ as the most toxic for algal growth in the case of Pb and as the most effective in induction of cell proliferation in case of phytohormones. Therefore, following auxins: 0.01 µM IAA, 0.1 µM IBA and 0.1 µM PAA as well as cytokinins: 0.01 µM *t*Z, 0.1 µM Kin, and 1 µM DPU were used solely and in combination with 100 µM Pb applied as nitrate form Pb(NO_3_)_2_. All exogenous compounds used in experiments were purchased from Sigma-Aldrich Co., USA. The parameters were analyzed on the 5th day of the experiment when algal cultures reached the maximal metabolic and mitotic activity as previously determined^3,4,9,48^.

### Phytohormone analysis

The method of phytohormone purification and determination described by Šimura *et al*.^51^ was employed. Algal samples (100 mg) were collected by centrifugation (4 °C, 10 min, 10,000*g*). A mixture of stable, isotopic labeled internal standards (IS): [13C_5_]-*t*Z, [2H_5_]-*t*ZR, [2H_5_]-*t*Z9G, [2H_5_]-*t*Z7G, [2H_5_]-*t*ZOG, [2H_5_]-*t*ZROG, [2H_3_]-DHZ, [2H_3_]-DHZR, [2H_3_]-DHZ7G, [2H_3_]-DHZ9G, [13C_5_]-*c*Z, [2H_5_]-*c*ZR, [2H_5_]-*c*ZOG, [2H_5_]-*c*ZROG, [2H_6_]-iP, [2H_6_]-iPR, [2H_6_]-iP7G, [13C_6_]-IAA, [2H_6_]-ABA, [2H_2_]-GA_3_ (1 pmol per standard) was added^5–8^. The calibration curves were constructed using serially diluted phytohormone standards and the labelled IS. Phytohormones were extracted from frozen algal cells using 1 mL ice-cold 50% aqueous (v/v) acetonitrile (ACN) in a bead mill (The TissueLyser LT QIAGEN, Germany) with two zirconium balls operating at a frequency of 50 Hz for 5 min. Samples were then sonicated (3 min, 4 °C), shaken in Eppendorf ThermoMixer (30 min, 4 °C) and centrifuged (10 min, 36,670*g*, 4 °C). The obtained supernatant was purified using Oasis HLB RP polymer-based SPE cartridges (Waters). Before purification, the cartridges were washedwith 100% methanol (MeOH) and deionized H_2_O and then equilibrated with 50% aqueous (v/v) ACN. After loading a supernatant, the flow-through fraction was collected in a glass tube. The cartridge was then rinsed with 30% (v/v) ACN, and this fraction was also collected in the same glass tube. The samples were evaporated to dryness under stream of nitrogen in an evaporation system, dissolved in 30% ACN (v/v), transferred to insert-equipped vials, and injected (10 µL) into the Agilent 1260 Infinity series HPLC system consisting of a degasser, binary pump, autosampler, and column oven coupled to an Agilent 6540 UHD Accurate-Mass Q-TOF LC/MS mass spectrometer with Dual Agilent Jet Stream Electrospray Ionization (Dual AJS ESI) source. The analytical column XSelect C18 column (250 mm × 3.0 mm, 5 μm) was heated up to 50 °C. Mobile phase A was 0.01% (v/v) formic acid (FA) in ACN and B 0.01% (v/v) FA in H2O, flow was 0.5 mL min^−1^. Separation of the above hormones was done in ESI positive mode with following gradients: 0–8 min flowing increased linearly from 5 to 30% A, 8–25 min 80% A, 25–28 min 100% A, 28–30 min 5% A. The parameters of MS were as follows: the nebulizer (nitrogen) gas pressure 60 psi, drying gas (nitrogen) pressure 50 psi, curtain gas pressure 30 psi, source voltage 3.5 kV and source temperature 350 °C.

### GC-MS analysis of intracellular metabolite profile

Molecular low weigh metabolites were extracted from algal cells using MeOH, then derivatized with *bis*(trimethylsilyl)trifluoroacetamide to gain trimethylsilyl derivatives and analyzed using a gas chromatograph (7890B GC System) with mass selective detector MSD 5977A (Agilent Technologies, USA) equipped with HP-5MS fused silica column (30 m × 0.25 mm × 0.25 μm)^3^. Metabolites were identified using an automatic system of processing data (Agilent MassHunter Workstation Software Qualitative Analysis) supplied by the National Institute of Standards and Technology (NIST) database.

### Determination of phytochelatins and their precursors

For Cys, *γ*-Glu-Cys, GHS and PCs analysis, the suspension of algal cells (500 mg) were centrifuged (4 °C, 10 min at 5,000g) and homogenized in 0.1% trifluoroacetic acid (TFA) (w/v) with 6.3 mM diethylenetriaminepentaacetic acid using a bead mill (The TissueLyser LT, QIAGEN, Germany)^29^. Thiol compounds were determined using reversed-phase HPLC with Fluorescence Light Detector (Agilent 1260 Infinity Series, USA) after derivatization with monobromobimane (mBBr) as fluorescent tag following the procedure described by Le Faucheur *et al*.^28^. Samples were separated on a COSMOSIL Packed Column 5C-18-MS-II (4.6 μm × 250 mm) (Nacalai, USA) at 37 °C. The mobile phase was MeOH and H2O, both containing 0.1% (v/v) TFA. A Linear gradient from 12% to 100% MeOH was used during 70 min of analysis. The retention times of the Cys, *γ*-Glu-Cys, GSH, and PC oligomers (PC_2_, PC_3_, PC_4_, and PC_5_) were verified by their standards (AnaSpec, EGT Corporate, USA). For the quantification of non-protein thiols, the calibration curves relating concentrations of standard solutions and the resulting peak area were used. The analytical data were integrated using ChemStation software for LC systems. Average synthesis rate of GSH was estimated using the calculation *C*_*t*_ =  *C*_0_ × *eµt*; where *C*_*t*_  =  GSH content at time t; *C*_0_  =  GSH initial content of GSH; *µ*  =  average synthesis rate, *t*  =  time.

### Phytochelatin synthase activity assay

Phytochelatin synthase (PCS) activity was determined according to a modified protocol by Finkemeier *et al*.^52^. The enzyme was extracted from algal cells in buffer containing 20 mM HEPES-NaOH, pH 7.5, 10 mM *β*-mercaptoethanol, 100 µM Pb(NO_3_)_2_, 20% (w/v) glycerol and polyvinylpyrrolidone (100 mg mL^−1^). The assay contained extract (400 µL), reaction buffer (25 mM GSH, 100 µM Pb(NO3)2, 10% (w/v) glycerol, 250 mM HEPES-NaOH, pH 8.0) and protease inhibitor. The samples were incubated for 90 min at 35 °C and terminated by the addition of 20% (w/v) trichloroacetic acid. Thiol groups were derivatized using mBBr and HPLC analysis was performed as described above. One unit of PCS activity was assumed as the amount of PC_2_ (nmol) synthesized per mg of soluble protein per minute at 35 °C. The content of soluble proteins in algal extracts prepared for the determination of PCS activity was measured following the Bradford^53^ method.

### Estimation of Pb content

The contents of Pb in *A. obliquus* cells treated with auxins and cytokinins were analyzed for estimation of the relation between Pb content and the level of the sum of non-protein thiols ∑SHtot = [GSH]+[2 × PC_2_+3 × PC_3_+4 × PC_4_+5 × PC_5_]. Pb level in algal cells was determined by flame atomic absorption spectrometry using a Solaar M6 (Thermo Electron Corporation, UK) spectrometer with a deuterium background correction system in air-acetylene flame (0.5 nm spectral bandpass, λ = 217.0 nm)^3^.

### Replication and statistical analysis

Each measurement consisted of 4 replicates, and each experiment was carried out at least twice at different times. Before selecting the appropriate statistical analysis method, the data were tested for normality (Shapiro-Wilk test) and homogeneity of variances (Levene’s test). The normality of data and homogeneity of variances were reported. Further, no significant outliers were found in data. Therefore, the data were analyzed using F-test and one-way ANOVA to establish statistically significant differences between calculated arithmetic means. The means were grouped using Tukey’s Post-hoc test (TIBCO Software Statistica version 13.3). The level of significance in all statistical tests and comparisons was *p* < 0.05^4,9^.

The R software^54^ and ‘FactoMineR’ package^55^ were used to perform principal component analysis (PCA) to uncover the phytohormone profile. Data were scaled to unit variance, and the first two factors, which explain about 86.47% of the variance, were selected based on percentage of explained variance criteria (Table 3).

**Table 3:** Eigenvalues and variances from PCA.

Dimension	Eigenvalue	Percentage of variance	Cumulative percentage of variance
Dim 1	15.940	72.456	72.456
Dim 2	3.081	14.006	86.462
Dim 3	1.025	4.660	91.122
Dim 4	0.724	3.292	94.414
Dim 5	0.547	2.487	96.901
Dim 6	0.537	2.439	99.339
Dim 7	0.145	0.661	100.000
